# Colour and Texture Descriptors for Visual Recognition: A Historical Overview

**DOI:** 10.3390/jimaging7110245

**Published:** 2021-11-19

**Authors:** Francesco Bianconi, Antonio Fernández, Fabrizio Smeraldi, Giulia Pascoletti

**Affiliations:** 1 Department of Engineering, Università degli Studi di Perugia, Via Goffredo Duranti 93, 06135 Perugia, Italy; 2 School of Industrial Engineering, Universidade de Vigo, Rúa Maxwell s/n, 36310 Vigo, Spain; antfdez@uvigo.es; 3 School of Electronic Engineering and Computer Science, Queen Mary University of London, Mile End Road, London E1 4NS, UK; f.smeraldi@qmul.ac.uk; 4 Department of Mechanical and Aerospace Engineering, Politecnico di Torino, Corso Duca degli Abruzzi 24, 10129 Torino, Italy; giulia.pascoletti@polito.it

**Keywords:** texture, colour, visual recognition, deep learning

## Abstract

Colour and texture are two perceptual stimuli that determine, to a great extent, the appearance of objects, materials and scenes. The ability to process texture and colour is a fundamental skill in humans as well as in animals; therefore, reproducing such capacity in artificial (‘intelligent’) systems has attracted considerable research attention since the early 70s. Whereas the main approach to the problem was essentially theory-driven (‘hand-crafted’) up to not long ago, in recent years the focus has moved towards data-driven solutions (deep learning). In this overview we retrace the key ideas and methods that have accompanied the evolution of colour and texture analysis over the last five decades, from the ‘early years’ to convolutional networks. Specifically, we review geometric, differential, statistical and rank-based approaches. Advantages and disadvantages of traditional methods vs. deep learning are also critically discussed, including a perspective on which traditional methods have already been subsumed by deep learning or would be feasible to integrate in a data-driven approach.

## 1. Introduction

The Psychology Dictionary gives two definitions of *visual recognition*: (a) ‘the capacity to identify an item visually’ and (b) ‘the ability to recognize items in their visual environment’ [1]; while *visual*, according to the Oxford English Dictionary, means ‘related to seeing or sight’ [2]. Colour and texture play a central role in this context: the ability to process these stimuli is one of the fundamental skills that enable humans to interact effectively with the environment they live in. Reproducing this capacity in artificial systems has therefore been a hot topic in computer vision since early on. Applications of colour and texture descriptors span many and very diverse areas, such as industrial inspection, remote sensing, medical image analysis, astronomy, autonomous systems, biometric identification, forensics, arts and cultural heritage.

Research in computer vision has been through major changes in recent years. Whereas theory-driven (also referred to as ‘classic’, ‘traditional’, ‘engineered’, ‘hand-crafted’, or ‘hand-designed’) approaches were the leading strategy no earlier than few years ago, data-driven methods (deep learning) are nowadays the main focus. Colour and texture analysis of course has not been immune to these changes. The objective of this note is to review the main concepts behind colour and texture descriptors from a historical perspective. We do not pretend, of course, to provide an exhaustive and comprehensive review of the subject: any such attempt would inevitably be bound to fail, given the huge number of methods that exist in the literature. Instead, we want to retrace the key ideas that have accompanied the evolution of colour and texture analysis over the last fifty years. By choice we decided to focus on the main concepts and not on equations and technicalities: the interested reader will find further details and the mathematical formulations in the cited references. Our approach is diachronic and synchronic at the same time: while our perspective is mostly historical, we also provide a taxonomy of the available methods and underline the key differences among them.

We clarify that this paper treats colour description from the perspective of image classification, hence neither colorimetry or colour science are discussed here: the reader interested in this subject will find extensive coverage in [3,4]. Likewise, texture analysis of infra-red and/or multi-spectral images is not in the scope of the present work: again we refer the reader to refs. [5,6,7,8,9] for an overview on this topic.

In the remainder we first give definitions of colour and texture, present a taxonomy of the descriptors and propose a historical periodisation (Section 2). For each time-frame we summarise the key ideas and overview the most prominent methods (Section 3, Section 4 and Section 5). We conclude the paper with a discussion about theory- vs. data-driven approaches, a much debated epistemic issue whose boundaries extends well beyond computer vision (Section 6 and Section 7).

## 2. Colour and Texture Descriptors for Visual Recognition: Definitions, Taxonomy and Periodisation

### 2.1. Colour and Texture

Neither colour or texture are easily defined concepts. However, while standard procedures exist for measuring colour, that doesn’t hold true for texture. Bigun speaks of colour as ‘the result of our cerebral activity, which constructs it from further processing of the photoreceptor signals’ ([10], p. 21). Although this might sound a bit vague, colour scientists have long agreed upon standards which enable colour to be specified quantitatively. The Commission Internationale de l’Éclairage (CIE) colorimetric system defines colour coordinates in such a way that (a) under the same observing conditions stimuli with the same coordinates are perceived as equal by an observer with normal colour vision; (b) stimuli that are perceived as equal have the same coordinates, and (c) the colour coordinates are continuous functions of the physical parameters of the light stimulus [3].

Texture, on the other hand, is related to the variation of the visual stimulus in a spatial domain. Therefore, unlike colour, texture cannot exist on a point, but needs an area large enough for the variation to be perceived. Various authors have proposed different definitions for texture, some of which are reported in Table 1. As can be seen, concepts commonly linked to the idea of texture are the presence of repeated patterns, the dependence on scale, variability and stationarity.

### 2.2. Taxonomy

Various classification schemes for colour and texture descriptors have been proposed in the literature [15,16,17,18,19,20,21]. In particular, [15,16,17,18,19] are classic taxonomies of texture descriptors for grey-scale images, whereas [20,21] also consider colour. All these works, albeit inevitably outdated, are very important from a historical perspective. More recent contributions to the field are the notable works of Liu et al. [22,23] and Humeau-Heurtier [24], both of which are again focused on texture.

**Figure 1:**
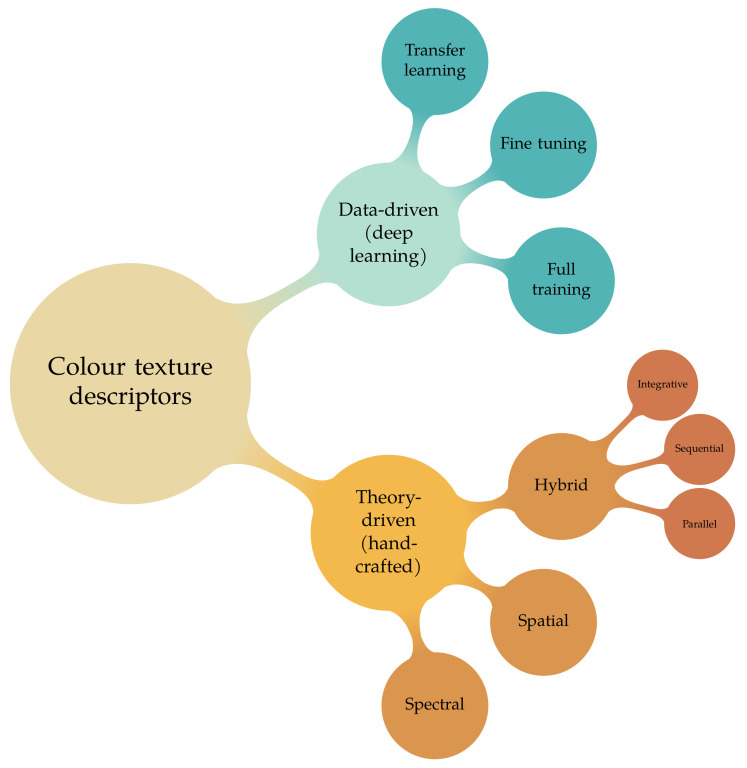
Colour texture descriptors: proposed taxonomy.

In this work we will follow the classification proposed in [25]: this embraces texture and colour descriptors as well as traditional and deep learning methods (Figure 1). This taxonomy identifies two main classes: the *theory-driven* approaches (also referred to as *traditional*, *hand-designed*, *hand-crafted* or *engineered* methods) and the *data-driven* ones, that is, deep learning. The methods of the first group are based on custom, hand-designed, mathematical functions which transform the input data (images) into sets of features, hence the term *feature engineering*. Such functions usually reflect some kind of perceptual, statistical and/or information-theoretical model. The hand-crafted methods are for most of their parts established a priori and require little or no training. On the other hand, the data-driven methods rely on computational blocks (*layers*) which contain a large number of free parameters the values of which are determined by training. As a result, the feature engineering process is mostly delegated to the training step. Of course there is still some “manual” work involved, but this is mostly limited to the design of the layers, their connections and the training procedure. The theory-driven/data-driven dichotomy is central here, and we shall return to it again later. For now, we refer the reader to ([26], Ch. 1) for an introduction to the topic.

On the hand-crafted side we have *spectral*, *spatial* and *hybrid* methods [27]. Spectral methods, also known as *colour* descriptors, take into account the colour content of an image but with no reference to its spatial distribution. As a result, these methods are fairly robust to geometric transformations (such as rotation, scale and/or viewpoint variations) but sensitive to changes in the illumination conditions. Spatial methods, on the other hand, consider the spatial variation of the image intensity but discard colour. These are traditionally referred to as (*grey-scale*) *texture* descriptors. Their characteristics are quite opposite to that of the spectral methods: they are in fact sensitive to geometric transformations, but to some extent resilient to changes in the illumination conditions. Finally, hybrid methods combine colour and texture together in different ways. Traditionally, these approaches have been categorised into three groups: *parallel*, *sequential* and *integrative* [20]. In parallel methods texture and colour features are extracted separately then concatenated (early fusion). In sequential approaches the input colour image is first converted into monochrome, then processed via some spatial descriptor. Integrative solutions obtain features by processing each colour channel separately and/or tuples of channels together. The latter step is usually achieved via some ad-hoc modifications of standard texture descriptors.

### 2.3. Periodisation

**Figure 2:**
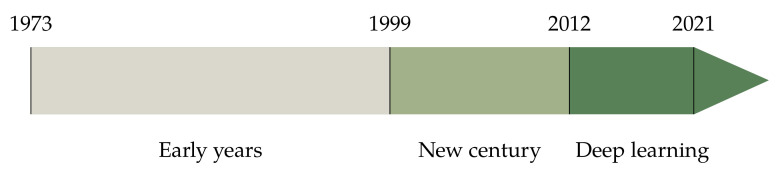
Colour and texture descriptors for visual recognition: overall periodisation.

**Figure 3:**
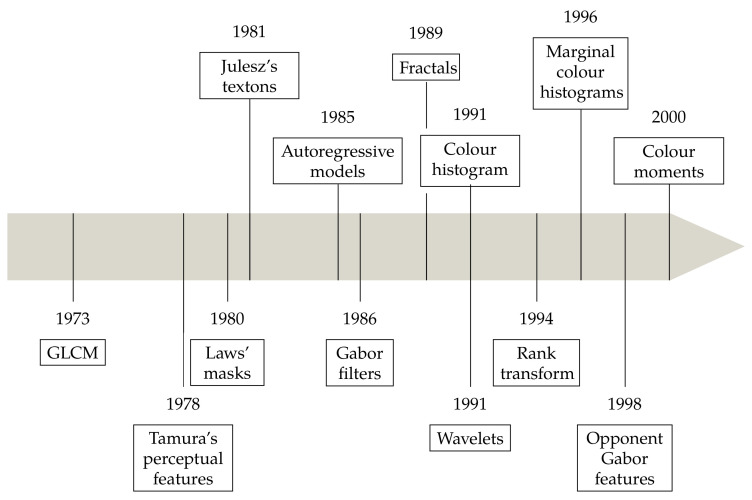
Colour and texture descriptors for visual recognition: chronology of the early years.

Categorizing historical developments into discrete blocks of time inevitably entails some degree of arbitrariness. Our periodisation starts with Haralick’s seminal work on co-occurrence matrices [28], published in 1973. Though this was not, strictly speaking, the first paper on computational methods for texture (Haralick himself mentions previous studies), its impact on the literature was profound. At the other end of the timeline, few would object that Krizhevsky et al.’s paper [29] (2012) on deep convolutional neural networks represented a major breakthrough in the field, that we will consider as the inauguration of the data-driven era. During the 42 years between [28,29], colour texture analysis attracted increasing attention and the number of descriptors grew constantly. It is not as easy to identify clear watersheds in this time frame; still, we believe that one major achievement was the formalisation of the bag-of-visual-words (BoVW) model thank to the works of Malik et al. [30,31], and Ojala et al. [32] at the turn of the century (1999–2002). We therefore identified three main periods in our chronology (Figure 2): *the early years* (Figure 3, *the new century* (Figure 8) and the *deep learning era* (Figure 15). Of course this choice involves an element of arbitrariness, and other methods such as Swain and Ballard’s colour histogram [33], Gabor filters [34] or wavelets [35] could have been reasonable milestones for different periodisations.

## 3. The Early Years

Looking back at it now, one could get the impression that colour and texture descriptors evolved quite erratically during the early years. This is largely true: indeed a lot of methods were proposed, most of them apparently unrelated to one another. Nonetheless, it is still possible to identify some leitmotifs that dominated the scene during this period. As regards spatial (texture) descriptors the dominant ideas were: (1) the *statistical* analysis of grey-level distributions as for instance implemented by co-occurrence matrices and related methods (Section 3.1.1); (2) image *filtering* (Section 3.1.5) aimed at capturing texture characteristics at different scales and orientations; and (3) autoregressive models for texture synthesis and reconstruction (Section 3.1.3).

For most of the early years there was more interest in texture than colour descriptors. The first work on colour analysis (Swain and Ballard’s colour histogram [33]) only came in 1991—that is, eighteen years later than the first work on texture. Also note that colour and texture were, at the beginning, dealt with separately. That changed after the appearance of opponent Gabor features, introduced by Jain and Healey in 1998 [36].

### 3.1. Spatial Descriptors

#### 3.1.1. Grey-Level Co-Occurrence Matrices

**Figure 4:**
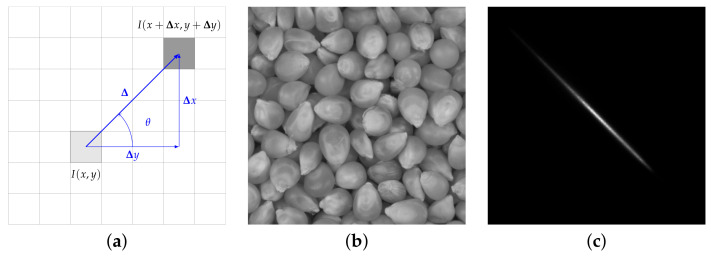
Grey-level co-occurrence matrices. From left to right: (**a**) mathematical formulation, (**b**) sample image and (**c**) corresponding GLCM ($\delta =3\sqrt{2}$ px, $\theta =45^{\circ }$). In (**a**) each square of the grid represents one pixel; (x,y) indicates the coordinates and I(x,y) the grey-level intensity. Sample image (**b**) sourced from RawFooT DB [45,46].

We open this overview with grey-scale co-occurrence matrices (GLCM), first introduced by Haralick in 1973 [28]. A GLCM is the two-dimensional $N_{g}\times N_{g}$ joint probability distribution of the intensity of pairs of pixels separated by a given displacement vector Δ(Δx,Δy), where $N_{g}$ indicates the number of grey-levels (Figure 4). For multi-scale and multi-directional analysis a set of co-occurrence matrices at different scales (δ) and orientations (θ) are normally computed, being δ and θ the polar coordinates (modulus and argument) of Δ. Co-occurrence matrices are not used directly as features; instead, the standard approach is to extract from them a few global statistical parameters such as *contrast*, *correlation*, *energy*, *entropy*, etc.

Co-occurrence matrices are a versatile, compact, yet effective method for texture analysis. They are used still today in different areas of computer vision, in particular medical image analysis [37,38,39,40]. The impact of GLCM on the literature was huge, and they directly inspired several other methods such as grey-level run lengths [41], grey-level differences [42], grey-level dependence matrices [42], neighbouring grey-level dependence matrices [43] and neighbouring grey-tone difference matrices [44].

#### 3.1.2. Tamura’s Perceptual Features

Tamura’s features were an attempt to describe texture through intuitive, human-interpretable concepts. To this end Tamura and colleagues [47] identified six such concepts, each corresponding to one texture feature. More precisely, Tamura’s descriptors comprise four ‘primary’ and two ‘secondary’ features, the latter being defined as linear combinations of the primary ones. The primary features are (1) *coarseness*, which is related to the size of the texture micro-patterns; (2) *contrast*, which depends on a combination of dynamic range (spread of the intensity histogram), polarisation, sharpness of edges and spatial period of the texture micro-patterns; (3) *directionality*, which reflects the tendency of the texture to show preferential directions or not and (4) *line-likeliness*, which indicates the prevalence of thin, elongated micro-patterns versus chunky, blob-like ones. The secondary features are (5) *regularity*, a function of the standard deviation of each of the four primary features and (6) *roughness*, defined as the sum of contrast and coarseness.

Tamura et al.’s attempt to describe textures via human-interpretable, intuitive, linguistic labels represents an original approach in the history of texture analysis. Although the impact of the method on other visual descriptors has been possibly limited (as discussed later, the general trend has been towards less and less human-interpretable descriptors), the approach has received attention in tasks like aesthetic perception of textures [48,49] and content-based image retrieval by linguistic queries [50].

#### 3.1.3. Autoregressive Models

Autoregressive models were originally proposed as a tool for texture synthesis [51]. Therefore, instead of just analysing textures, autoregressive models seek a way for representing and reproducing them. They are based on the assumption that the relation between the pixels’ intensities within a given neighbourhood can be modelled through some suitable mathematical functions—typically a linear combination of Gaussians. The parameters of the model (weights) are determined by fitting to the given texture. The rationale behind the method is that the parameters of the model in fact represent intrinsic characteristics of the textures, and can be therefore used as texture features [52].

#### 3.1.4. Fractals

Fractal models are based on the concept of self-similarity. A set in a Euclidean *n*-space is said to be self-similar when it is the union of distinct copies of itself, the copies being scaled down by the same reduction factor. The number of copies and the scale factor determine the fractal dimension of the set through a well-known formula ([53], Equation (1). In the traditional approach the input image is treated as an elevation model $\left\{x,y,z=I\left(x,y\right)\right\}$; the procedure then estimates, for each (x,y), the number of points within a cube of side *L* as a function of *L* (*box counting*). In the last step, some global parameters are computed from the resulting function, as for instance slope, intercept and lacunarity. Since the seminal work by Keller [53] the method has been extended in various directions, including other strategies for feature extraction [54,55] and colour images [56].

#### 3.1.5. Filtering

Filtering was a dominant idea in the early years of texture analysis. The overall procedure is very general and consists of the following steps: (1) design of a bank of filters, (2) convolution of the input image with the filters and (3) extraction of global statistics from the transformed images—e.g., average, energy and/or standard deviation. The texture features are eventually obtained by concatenating the parameters extracted from each of the transformed images. There can be further post-processing to achieve some desired properties such as contrast normalisation and/or invariance against rotation. The differences between the various methods lie in the types of filter used. Here we recall three classes of filters that have had particular importance from a historical perspective: Laws’ masks, Gabor filters and wavelets. For a comprehensive review of filtering in the early years we also recommend the classic work of Randen and Husøy [57].

##### Laws’ Masks

**Figure 5:**
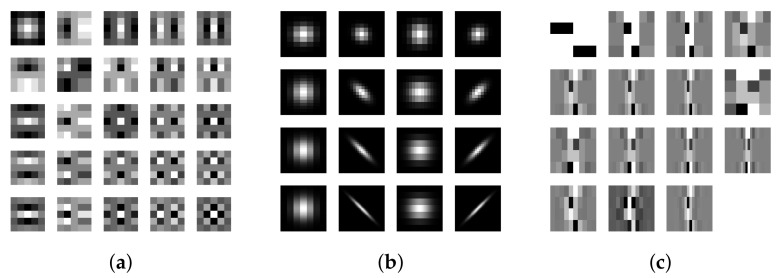
Samples of filter banks: Laws’ masks (**a**), Gabor filters (**b**) and bi-orthogonal wavelets (**c**).

Filtering for texture analysis was first introduced by Laws in 1980 [58]. For this task he proposed a bank of twenty-five 5 px × 5 px separable linear filters (later on referred to as Laws’ masks) generated by combining five one-dimensional filters (Figure 5a). This formulation makes Laws’ masks computationally fast, a very much appreciated feature in times when computing power was a tiny fraction of what is today.

##### Gabor Filters

Gabor filters are a class of orientation- and frequency-selective steerable filters (Figure 5b). For a long time they were considered the most effective filtering method for extracting textural features [19]. Their use is motivated by perceptual and mathematical considerations. Perceptually, they are believed to be a good approximation for the receptive field of the simple cells in the V1 and V2 visual cortices [59,60]. Mathematically, Gabor filters have optimal joint resolution in the two-dimensional space and frequency domain [59,60]. Experiments of texture analysis with Gabor filters date back to 1986 with the work of Turner, followed by others soon thereafter [61,62].

##### Wavelets

Wavelets overcome one inconvenience common to some signal transforms (such as the Fast Fourier Transform), that is, fixed resolution in the spatial and frequency domains. Grossmann and Morlet [63] introduced wavelets as families of functions obtained from one single function (the ‘mother’ wavelet) by dilations and translations. The mother wavelet is a zero-mean, rapidly decaying oscillation, that characterises each specific family of wavelets. Texture classification by wavelets was first investigated by Carter [64], who proposed the use of Mexican hat and Morlet wavelets. Other families of wavelets (including Daubechies, Haar, orthogonal and bi-orthogonal) have also been used in later works [65,66,67].

### 3.2. Julesz’s Textons

In 1981 Julesz penned a very influential paper on the perception of textures [68]. His main claim was that texture perception is the result of the combination of local, elementary texture units (‘elongated blobs of specific widths, orientations and aspect ratios’) which he referred to as *textons*. He also suggested that only the first-order distribution of such elements is relevant to pre-attentive identification and discrimination of textures, whereas higher-order differences can only be perceived after detailed scrutiny. Julesz did not actually define any computational method for texture analysis, but his hypothesis represented the rationale of the BoVW model, which came to a complete formalisation two decades later (see Section 4.1).

### 3.3. Rank Transform

**Figure 6:**
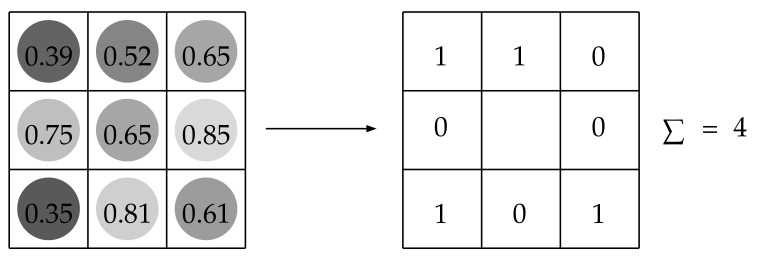
The rank transform.

Given a centre-symmetric neighbourhood of pixels, the rank transform (RT) considers the number of pixels having grey-value less than that of the central pixel [69] (Figure 6). Although the RT was originally introduced for visual correspondence, it has historical relevance to texture analysis in that it is the first method based on local comparisons between grey-scale intensity values. This concept inspired other approaches to texture based on non-parametric statistics, such as Ranklets and Local Binary Patterns (more on this in Section 4). The main advantages of the rank transform are ease of implementation, low dimensionality and little computational demand—all of which make the method particularly suitable for real-time applications [70,71].

### 3.4. Spectral Methods

#### 3.4.1. Colour Histogram

**Figure 7:**
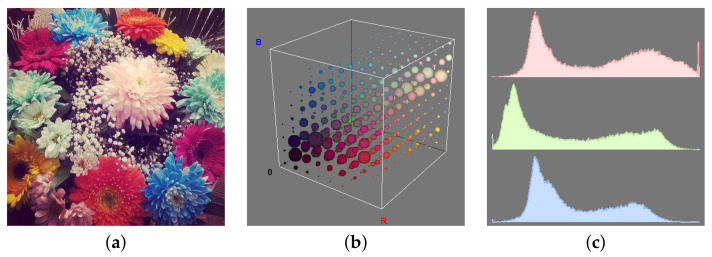
Colour histograms in the RGB space. From left to right: sample image (**a**), three-dimensional colour histogram (**b**) and marginal histograms (**c**). Sample image (**a**) sourced from Wikimedia Commons (https://upload.wikimedia.org/wikipedia/commons/thumb/d/dc/Bunch_of_flowers_.jpg/640px-Bunch_of_flowers_.jpg accessed on 7 June 2021) (CC BY-SA 3.0).

Central to the spectral (colour) descriptors of the early years is the idea of colour histogram. Given a colour space and a discrete set of predefined colours in that space (the *palette*) the colour histogram (also referred to as ‘full’ or ‘joint’ colour histogram [25]) records the occurrence probability of each colour of the palette in the input image (Figure 7a,b). The palette can be either established a priori—typically by uniform or non-uniform quantisation of each axis of the colour space; or a posteriori, via some clustering procedure. In their seminal work Swain and Ballard [33] used non-uniform quantisation of the opponent colour space (*black-white*, *red-green* and *blue-yellow*) into respectively 8, 16 and 16 levels for each channel. Another common implementation is uniform quantisation of the RGB space, as for instance used in [25,72,73]. Despite its conceptual simplicity, various studies have demonstrated the effectiveness of the colour histogram for discriminating colour textures under steady illumination conditions [25,72]. One potential drawback, however, is the number of features the method generates. Denoting as $N_{g_{c}}$ the number of quantisation levels for the *c*-th colour channel, the number of features is $f=\prod_{c=1}^{C}N_{g_{c}}$; that is, the dimensionality grows as the *C*-th power of the number of quantisation levels per channel, where *C* denotes the dimension of the colour space (usually C=3).

#### 3.4.2. Marginal Histograms

Marginal histograms are the concatenated one-dimensional distributions of the intensity levels of each colour channel, or, in other words, the marginalised version of the colour histogram (Figure 7a,c). One clear advantage of marginal histograms versus colour histogram is the lower dimensionality: in this case the number of features $f=\sum_{c=1}^{C}N_{g_{c}}$ grows linearly with the number of quantisation levels. Compared with colour histogram, this usually comes at the cost of a slightly inferior discrimination capability for colour textures [25,72].

#### 3.4.3. Colour Moments

Colour moments are closely related to full and marginal colour histograms; however, instead of using the complete colour distributions directly, a set of moments is computed and these are used as image features. Although it is common to present colour moments as by-products of colour histograms [74,75], it is worth recalling that moments can be actually computed independently from histograms. In Paschos’s implementation [74] the moments were obtained from the two-dimensional probability distribution on the chromaticity diagram $\left(\frac{X}{X+Y+Z},\frac{Y}{X+Y+Z}\right)$ of the CIE XYZ colour space—hence the name (*chromaticity moments*). However, the method is easily generalised to any colour space and combination of colour axes [25,75,76,77].

### 3.5. Hybrid Methods

#### Opponent Gabor Features

Jain and Healey [36] proposed an extension of Gabor filters to colour images based on intra- and inter-channel analysis. The intra-channel part consists of computing the features from each colour channel separately as described in Section 3.1.5, whereas inter-channel (opponent) features are obtained from the difference between the normalized transforms of pairs of colour channels, similar to what we described in Section 3.4.1. This idea of cross-channel analysis was later on transposed into other methods such as integrative co-occurrence matrices and opponent-colour local binary patterns (Section 4.3).

## 4. The New Century

**Figure 8:**
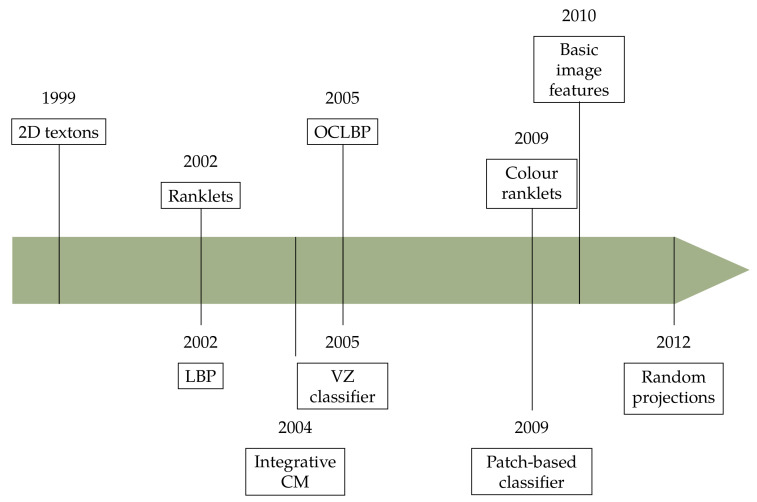
Colour and texture descriptors for visual recognition: chronology of the new century.

In the new century (Figure 8) colour and descriptors evolved along more clearly identifiable lines. In particular, the bag-of-visual-words model was the dominant paradigm in this period. Combined analysis of colour and texture also came to a clear formalisation thank to the work of Palm [20], to whom we owe the classification into parallel, sequential and integrative methods, which is by and large still valid today. The relative importance of colour and texture in colour texture descriptors was also investigated in a number of studies [21,72,78].

### 4.1. The Bag-of-Visual-Words Model

**Figure 9:**
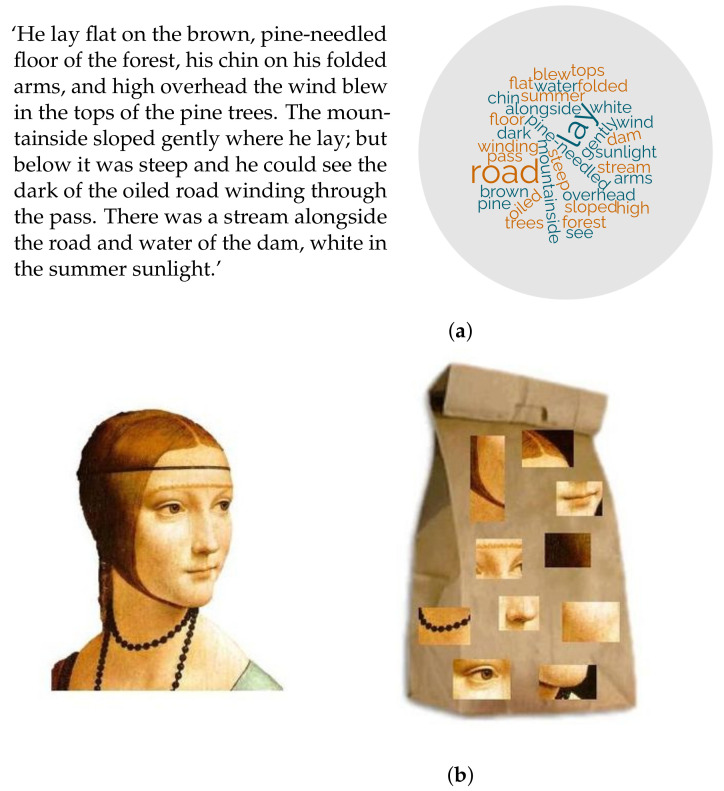
The BoW and BoVW models. (**a**) An illustration of the bag-of-words model: original text (**left**) and bag of words (**right**). Original text from ([79], p. 3); word cloud generated with https://www.wordclouds.com/ accessed on 9 November 2021. (**b**) An illustration of the bag-of-visual-words model: source image (**left**) and bag of visual words (**right**). Images sourced from Wikimedia Commons (https://upload.wikimedia.org/wikipedia/commons/thumb/0/08/Bag_of_words.JPG/640px-Bag_of_words.JPG accessed on 17 June 2021) (Public domain).

The bag-of-visual-words model is best explained by recurring to a parallel with its counterpart in natural language processing: the bag-of-words model (BoW). In the BoW a text is characterised through the frequency by which each word of a predefined set (the *dictionary*, D in the remainder) appears in the text, disregarding the word order. Likewise, the BoVW describes an image through the distribution probability of certain local image features (*visual words*) regardless of their spatial distribution (Figure 9).

This general scheme can be implemented in various ways, giving rise to different descriptors [80]. Specifically, there are two design choices which are of particular interest from a historical perspective: (1) the way the visual words are computed, and (2) how the dictionary is generated. As for the visual words, the common approach is to compute them through suitable functions which take as input the grey-levels or colour triplets of groups of pixels arranged in some spatial neighbourhood. The visual words can be extracted either from the image patches directly or from the local response (*jet*) of some filter bank. Regarding the dictionary, this can either be defined a priori (such as in Local Binary Patterns), or generated a posteriori (as for instance happens in the image patch-based classifier). Finally, the *pooling* (or *aggregation*) process—i.e., the estimation of the distribution of the visual words over the dictionary—can be implemented in different ways. Beyond the standard first-order statistic (histogram), other options are vectors of locally-aggregated descriptors (VLAD) and Fisher vectors (FV). For a thorough discussion on aggregation see also [81].

### 4.2. Spatial Methods

#### 4.2.1. BoVW

##### Two-Dimensional Textons

**Figure 10:**
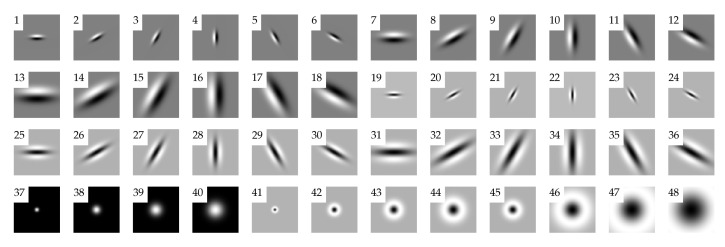
The ‘LM’ filter bank: even (1–18) and odd (19–36) Gaussian derivative filters at three scales (half-octave spacing) and six orientations (equal spacing from 0 to π), four Gaussian filters (37–40) and eight Laplacian of Gaussian filters (41–48).

In Section 3.1.5 we have had the chance to underline the fundamental role of filtering in the design of spatial (texture) descriptors. The usual approach of the early years consisted of computing the transformed images from each filter in the bank, extracting global statistics from each transformed image and concatenating the results into one feature vector. Malik et al. [30] took this idea one step further. They stacked the transformed images so that each pixel of the original image would be represented by a $N_{fil}$-dimensional vector, being $N_{fil}$ the number of filters in the bank. In their original model they used a bank of $N_{fil}=36$ Gaussian derivative filters at three scales and six orientations [30], and later on extended this number to 48 with the addition of 12 rotationally invariant filters [31]. The latter version came to be known as the ‘LM’ filter bank from the initials of its inventors (Figure 10). Then they obtained the dictionary of visual words (two-dimensional textons) by clustering the vectors into a set of prototype responses via *K*-means. Note that the size (cardinality) of the dictionary D is user-defined in this scheme and depends on the number of clusters (*K*) extracted from the train images. For instance in [31] the authors extracted four hundred visual words from each of the 20 training classes, which resulted in $\left|D\right|=400\times 20=8000$.

##### Local Binary Patterns

**Figure 11:**
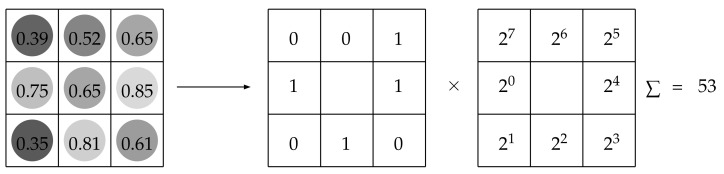
Local binary patterns.

Local binary patterns are an implementation of the BoVW in which the dictionary is defined a priori. For a centre-symmetric neighbourhood of pixels, the *kernel function* [82] of LBP compares the grey-value of each pixel in the periphery with that of the central pixel and assigns ‘1’ whenever the former is greater than or equal to the latter, ‘0’ otherwise [32]. The resulting binary string represents the unique code of the local image patch (Figure 11). Consequently, the size of the dictionary depends on the size of the neighbourhood: it is $\left|D\right|=2^{N_{p}}$ where $N_{p}$ is the number of peripheral pixels. The neighbourhoods are typically interpolated or non-interpolated (digital) circles of pixels [32,83], even though different arrangements have also been proposed [84,85].

Local binary patterns have been possibly one of the most successful texture descriptors of the hand-designed era, and they are still largely used to this day. The fortune of this methods relies a great deal on its conceptual simplicity, ease of implementation, low computational demand and high discrimination capability. The impact on the literature was also enormous: LBP inspired a huge number of variations which nowadays can be considered a class of their own (for comprehensive reviews on LBP variants see refs. [22,80,82,86]).

##### VZ Classifier

The VZ classifier (again named this way after its inventors—Varma and Zisserman [87]) builds on Malik et al.’s two-dimensional textons [30] with some minor modifications. Specifically, the filter bank used in the VZ classifier is very similar to LM’s, but employs 38 filters instead of 48; furthermore, the output of the oriented filters are made rotation-invariant by recording only the maximum filter response across all orientations. Another difference is that the input images are normalised to zero mean and unit variance, and that the filter responses at each pixel are contrast-normalised through Weber’s law.

##### Image Patch-Based Classifier

The image patch-based classifier (IPBC) is another implementation of the BoVW model [88]. The original idea here is that the visual words are the raw pixel intensities of local image patches, without these being processed through either filtering (as in two-dimensional textons or the VZ classifier) or via some kernel function (as in LBP). In the IPBC the dictionary of visual words is generated by clustering the raw pixel intensities of the local patches over a set of training images. The results reported in [88] demonstrate that the IPBC is as effective as BoVW implementations based on filter responses. This method represented a milestone in the development of traditional texture descriptors, since it demonstrated that multi-scale, multi-orientation filter banks were not strictly necessary. The major consequence was that the interest in hand-designed filter for texture analysis started to decline significantly after [88]. Nonetheless, as we shall see in Section 5, filters would come up again in deep learning, although, for a good part of them, no longer designed by hand.

##### Basic Image Features

**Figure 12:**
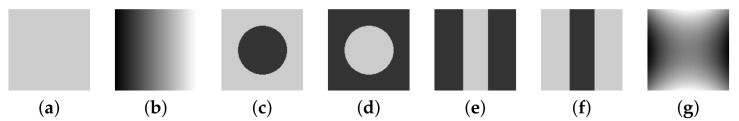
The seven archetypal image patches of the BIF: flat area (**a**), gradient (**b**), dark spot (**c**), bright spot (**d**), bright edge (**e**), dark edge (**f**) and saddle (**g**).

The Basic Image Features (BIF) employ an a priori dictionary of visual words based on local filter responses [89]. The dictionary is generated by partitioning the jet space into seven archetypes corresponding to different kinds of local image structures (Figure 12): *flat area*, *gradient*, *bright spot*, *dark spot*, *bright edge*, *dark edge* and *saddle*. Mathematically, each of these archetypes can be characterised through its invariant properties to one or more planar isometries. In particular, the flat area is invariant to all planar isometries, the gradient to a reflection about the axis of the gradient and a translation along an orthogonal axis, the spot to any rotation around the centre and any reflection about an axis passing through the centre, the edge to a translation along one axis and a reflection about an orthogonal axis, the saddle to a reflection about two pairs of orthogonal axes crossing at the centre of the patch.

##### Random Projections

Random projections (RP) are an attempt to overcome the dimensionality issues intrinsic to the IPBC and other BoVW models. In the IPBC, in particular, the size of the local neighbourhood determines the dimension of the VW space. For a local window as small as 3×3 the visual words are points in a nine-dimensional space, and the dimension grows quadratically with the side length of the local window. Consequently, the problem becomes quickly untreatable for large patches. The objective of random projections is to enable dealing with larger patches while maintaining the dimension of the VW space low. The solution proposed by Liu and Fieguth [90] is to project the original data into a lower-dimensional subspace while preserving the relative distance between any two points. This can be achieved through a linear mapping with the coefficients drawn from a Gaussian distribution, as for instance described in [90]. The authors showed that RP enables cutting the dimensionality of the problem down to one-third of that determined by the original patch without affecting the discrimination accuracy.

#### 4.2.2. Ranklets

**Figure 13:**
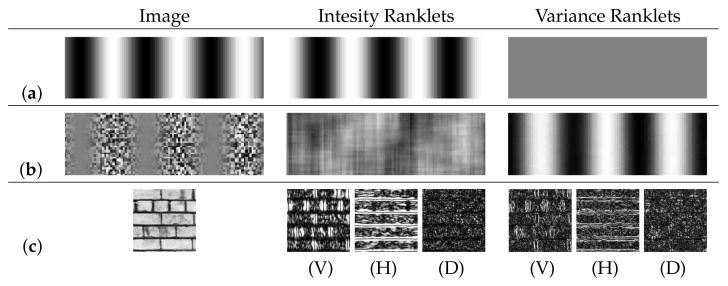
Intensity and Variance Ranklet responses to (**a**) an intensity modulation; (**b**) a variance modulation; (**c**) a texture image. For (**a**,**b**), vertical Ranklet responses are shown; for (**c**), we show the absolute value of Vertical, Horizontal and Diagonal filters. Image sources: texture D55, Brodatz [92,97].

Ranklets are a family of rank features that offer orientation selectivity patterns similar to Haar wavelets [91]. Given a Haar wavelet *h* with values in {+1,−1}, Ranklets compute the Mann-Whitney statistics for the comparison of the brightness of pixels in $h^{-1}\left(+1\right)$ with those in $h^{-1}\left(-1\right)$ (i.e., the two halves of the support window, taken horizontally, vertically or as pairs of opposite quadrants). This is equivalent to counting the number of pairs of pixels $\left(a,b\right)\in h^{-1}\left(+1\right)\times h^{-1}\left(-1\right)$ such that I(a)>I(b), where *I* indicates the pixel intensity. This operation, however, is done efficiently at the cost of a simple sorting operation. Ties are handled by the method of half-ranks. By replacing the Mann-Whitney statistics with the Siegel-Tukey statistics for dispersion (essentially a permutation of the ranks), Ranklets can be made to respond to second-order stimuli (variance modulations) [92]. Sample Ranklet responses are displayed in Figure 13. The extension of Ranklets to integrative colour descriptors was introduced in [93], where these features are computed separately on each colour channel and jointly on couples of channels. Besides grey-scale and colour image applications, Ranklets have been successfully applied to texture analysis in mammographic (X-ray) [94] and sonographic (ultrasound) [95] images of breast tissue and in computed tomography (CT) images of brain tissue [96].

### 4.3. Hybrid Methods

#### 4.3.1. Integrative Co-Occurrence Matrices

**Figure 14:**
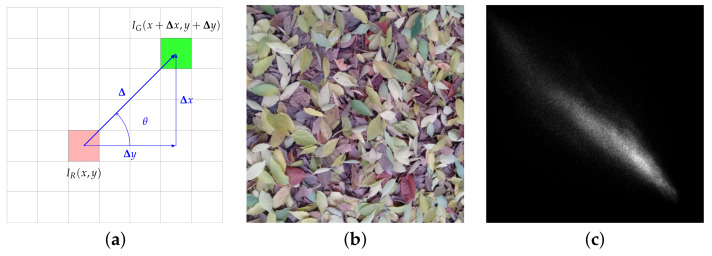
Integrative co-occurrence matrices. From left to right: (**a**) mathematical formulation, (**b**) sample image and (**c**) corresponding inter-class R-G co-occurrence matrix ($\delta =3\sqrt{2}$ px, $\theta =45^{\circ }$). In (**a**) each square of the grid represents one pixel; (x,y) indicates the coordinates, and $I_{R}\left(x,y\right)$, $I_{G}\left(x,y\right)$, respectively, the intensity in the red and green channel. Sample image (**b**) sourced from the T1K+ dataset [100].

Integrative co-occurrence matrices (ICM) generalise GLCM by considering co-occurrences of intensity values within each colour channel (intra-channel analysis) and between pairs of colour channels (inter-channel analysis). Intra-channel features are computed by extracting GLCM features from each colour channel separately; inter-channel features from pairs of colour channels jointly (Figure 14). Interestingly, the method appeared in two papers—both dated 2004—which apparently bear no relationship with one another [20,98]. Although ICM are usually computed on RGB images, other colour spaces such as HSV [98] and CIE Luv [20] have been investigated too. Custom implementations that take into account the image de-mosaicing process have also been proposed [99].

#### 4.3.2. Opponent-Colour Local Binary Patterns

Opponent-colour local binary patterns (OCLBP) are an extension of LBP to colour images [101]. Similarly to ICM the method extracts LBP features by processing each colour channel separately and pairs of colour channels jointly. The intra-channel analysis is performed by comparing the intensity of the peripheral pixels of the neighbourhood in one colour channel with the intensity of the central pixel in another colour channel. This scheme was original implemented in the RGB colour space using R/G, R/B and G/B as the opponent pairs for the inter-channel analysis [101]. As a result OCLBP generates six times the number of features produced by LBP. Comparative evaluations showed that OCLBP is generally better than LBP at discriminating colour textures under steady illumination conditions [25,102].

## 5. Deep Learning

We have seen in Section 3 and Section 4 that before deep learning colour and texture descriptors were mostly established a priori. We say ‘mostly’ because some of these methods do in fact require a certain amount of training: this is true, for instance, with some implementations of the BoVW such as two-dimensional textons, VZ classifier and IPBC (Section 4.1). This training part, which was marginal and limited to a small class of hand-designed descriptors becomes central in deep learning.

**Figure 15:**
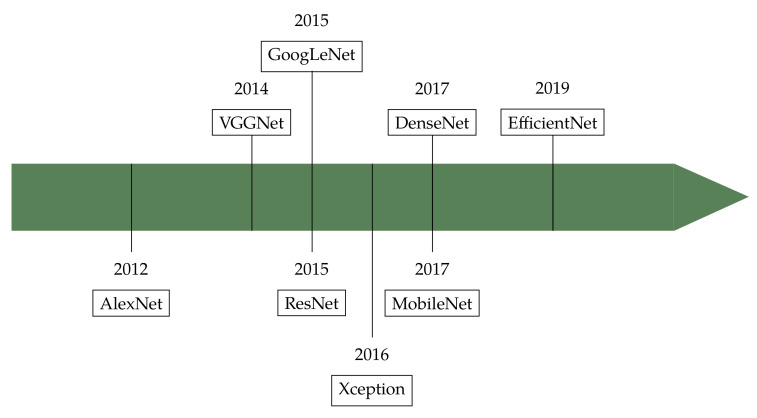
Colour and texture descriptors for visual recognition: deep learning.

Lecun et al. define deep learning as a set of representation-learning methods based on the composition of non-linear modules, each transforming the representation from one level into a slightly more abstract one [103]. In computer vision this idea finds its realisation in *convolutional neural networks* (CNN), which are generated by combining certain functional modules (*layers*) into complex structures. Some such layers (the *trainable* or *learnable* ones) contain large sets of parameters (*weights*) the values of which are not known a priori, but need to be determined by training. The training is an optimisation procedure which minimises the network misclassification error over a set of labelled examples. As a result the weights will incorporate the ‘knowledge’ acquired by the network consequent to the exposure to the train data. In the following subsections we briefly review the basic layer types (i.e., convolutional, pooling and fully-connected), the main CNN architectures (see also Figure 15 for a chronology chart) and discuss the usage for colour texture classification. For further details and technicalities on CNN we refer the reader to refs. [26,103,104,105].

### 5.1. Basic CNN Blocks

**Figure 16:**
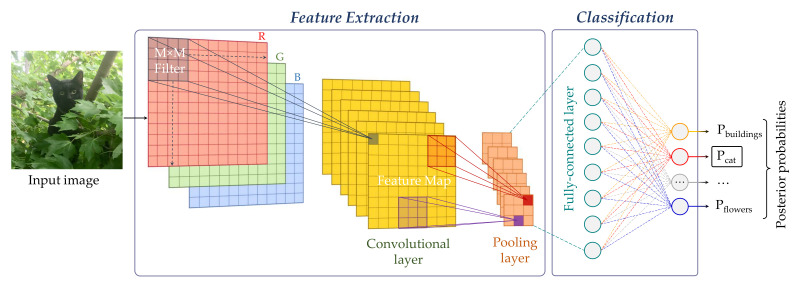
Sample architecture of a convolutional neural network.

The basic building blocks of a convolutional neural network are the convolutional, pooling and fully-connected layers [26,104]. Independently of the type, each layer transforms one multi-dimensional array into another multi-dimensional array, normally of different size. The input to the first layer of the network (*receptive field*) is usually a H×W×C matrix, the symbols, respectively, indicating the height, width and number of colour channels. In most cases the receptive field is square (H=W), and there are three colour channels (C=3). The output of the last layer is a one-dimensional array of *M* elements each representing the probability of one among the *M* possible classes the network has been trained on. In the classic configuration (Figure 16) the network is wired such as that the the output of one layer is the input to the following one, but as we shall see later other architectures also exist. Intuitively, we can say that a CNN progressively works the original image in a way that increases the third dimension (depth—colour channels) while reducing the other two (height and width).

#### 5.1.1. Convolutional Layers

Convolutional layers are banks of linear filters—therefore similar to those discussed in Section 3.1.5—but with two main differences. First, in a CNN the weights of the filters are not established a priori, but learnt; second, the convolutions operate on the spatial and chromatic level at the same time and in a way that reduces the spatial dimension of the input (width × height) while increasing the depth. Furthermore, whereas hand-designed filters are generally intuitive and their behaviour easy to predict, the functioning of convolutional layers becomes less and less interpretable as we proceed deeper into the network’s structure.

#### 5.1.2. Pooling Layers

Pooling layers reduce the dimension of the representation by sub-sampling the input at the spatial level. Differently from the convolutional layers, the transformation is hard-coded in this case and in general consists of replacing a group of pixel (usually a 2 × 2 window) by their maximum or average value (*max* and *average* pooling, respectively). Pooling layers achieve two main objectives: first, they reduce the number of coefficient to process; second, they make the downstream convolutional layers process increasingly large chunks of the original image, this way generating a hierarchical representation.

#### 5.1.3. Fully-Connected Layers

In fully-connected layers each element of the output field depends on all the elements of the input field. Fully-connected layers are usually the last components of the net. Ideally, they implement the top-level reasoning by combining the local image features they receive from by the upstream part of the network.

### 5.2. Architectures

The design of a CNN is a trade-off between two competing objectives: the demand for accuracy, which leads to adding layers and parameters; and the need to reduce the computational cost and limit overfitting problems, which requires limiting the number of layers and parameters. This reflects clearly in the historical evolution of CNN. Here below we briefly revise some of the most common architectures presenting in chronological order of appearance (Figure 15 and Table 2). We refer the reader to the given references for further details and technicalities (see also [106] for a recent survey).

#### 5.2.1. AlexNet

Altough this was not the first example of a CNN for computer vision (LeNet is a notable antecedent [113]), few would object that the appearance of AlexNet [29] marked a turning point in the field. This is mostly due to the success obtained in the ImageNet large scale visual recognition challenge (ILSVRC 2012), where the network attained a top-5 error of 15.3%, outperforming the second-best method by more than ten percentage points. The layout of AlexNet is fairly simple and consists of five convolutional, three pooling and three fully-connected layers for a total of ≈61 M trainable weights.

#### 5.2.2. VGGNet

The VGG16 and VGG19 models [107], developed within the Visual Geometry Group at the University of Oxford, United Kingdom, are conceptually very similar to the AlexNet but ‘deeper’—i.e., they feature a higher number of layers and parameters. Specifically, both models have five max pooling, three fully-connected and one soft-max layers, whereas the number of convolutional layers is 13 and 16, respectively, for the VGG16 and VGG19. On the whole there are ≈138 M trainable weights in the VGG16 and ≈144 M in the VGG19.

#### 5.2.3. GoogLeNet

The main innovation introduced with the GoogLeNet [108] is the ‘inception’ module, whose main objective is to avoid overfitting and reduce the computational overhead. Simply put, an inception module enables performing convolutions at different scales on the same level, so that the network gets progressively wider, not deeper. The resulting architecture is 27 layer deep, of which 22 are the learnable layers. The number of trainable weights is ≈6.7 M.

#### 5.2.4. Residual Networks (ResNets)

After AlexNet the overall trend was to increase the depth and/or the width of the network by respectively adding layers or increasing the size of the convolutional kernels. However, deeper networks are prone to overfitting and likely to incur the vanishing gradient problem: repeated multiplication can make the gradient very small as this is propagated to the upstream layers [114]. The main novelty with residual networks was the use of shortcut (‘skip’) connections to jump over one or more downstream layers [109]. Skip connections add the outputs from previous layers to the outputs of stacked layers; this tweak provides an ‘alternative path’ for the gradient and makes it possible to train very deep networks. Thank to this improvement it was possible to train convolutional networks up to 152-layer deep (ResNet 152, ≈60 M weights), that is, eight time deeper than VGG19.

#### 5.2.5. Densely Connected Networks (DenseNets)

We have seen in the previous paragraph how ResNets altered the classic structure of convolutional networks (Figure 16) by adding skip connections. DenseNets took this idea one step further by connecting each layer to every other downstream layer in a feed-forward fashion [110]. Furthermore, differently from ResNets, DenseNets do not add the output of a layer to the the incoming feature map but concatenate them. This architecture limits the vanishing-gradient and substantially reduces the number of parameters. This way it was possible to put together a network with more than 200 layers (DenseNet201) while keeping the number of trainable parameters relatively low (≈20 M).

#### 5.2.6. MobileNets

MobileNets are a kind of light-weight, computationally cheap networks designed for mobile and embedded vision applications (hence the name). The key concept behind this architecture is the use of depth-wise separable filters in the convolutional layers [111]. This breaks the interaction between the number of output channels of a layer and the size of the convolutional kernel, therefore reducing the total number of parameters and increasing speed. The original MobileNet features 28 layers with just above 4.2 M parameters.

#### 5.2.7. EfficientNets

EfficientNets represent a general solution to the size vs. accuracy dichotomy. This architecture relies on a resizing method that uniformly scales the depth, width and resolution of a network through a simple compound coefficient [112]. Starting from a mobile-size base architecture (EfficientNet-B0) the authors developed six up-scaled models (EfficientNet-B1, …, B7) which achieved comparable or better accuracy than previous methods with one-third to one-tenth fewer parameters.

### 5.3. Usage

Convolutional neural networks can be used in different ways, but the three main approaches are: *full training*, *fine tuning* and *transfer learning*. In full training the weights of all the learnable layers are initialised randomly and their final values determined via training. Since the number of parameters can be on the order of the tens of millions, full training requires dedicated hardware and is a time-consuming process. Furthermore, very large datasets are needed to avoid overfitting problems.

Transfer learning is the opposite strategy: here we take a network that has been trained for some specific task (e.g., object recognition, facial analysis or scene classification) and reuse it ‘as is’ for a different task. In this case it is customary to remove the fully connected layers at the end of the network (where the actual class predictions are made) and use the network as a feature extractor. This approach is also referred to as ‘off-the-shelf’ usage, and has proven surprisingly effective in many applications [115].

Finally, fine tuning is an intermediate solution between full training and transfer learning. The procedure consists of the following steps: (1) take a pre-trained network (the *backbone*), (2) reshape (if required) and re-initialise the weights of the last fully-connected layer (where the class predictions are made), (3) freeze the backbone and re-train the last fully-connected layer only, (4) optionally unfreeze some of the other layers of the backbone and retrain them [26]. The overall objective is to readjust the backbone network to make it more suitable for the problem at hand.

#### CNNs for Colour Texture Classification

During the last few years the use of convolutional networks for colour texture classification has received increasing attention. In particular, pre-trained networks used off-the-shelf have become very popular. This strategy has indeed several advantages: it is computationally cheap, relatively easy to implement and fairly effective. Furthermore, there are many pre-trained models one can choose from. In this approach one cannot of course use the output of the network directly, for the number and/or the types of classes the network was trained on will differ from those involved in the texture classification problem. Instead, it is customary to use the pre-trained network as a feature extractor, commonly by removing one or more of the last layers. This generates an ‘intermediate’ representation [45,100,116] which abstracts from the specific classes the network was trained on. When dealing with colour textures this can be implemented in two ways: one can either (a) generate *orderless* features by aggregating the output of a convolutional layer around a dictionary—therefore obtaining, to all extents and purposes, a BoVW representation [25,117,118]; or (b) extract *order-sensitive* features by taking the $L_{1}$ or $L_{2}$ normalised output of one of the fully-connected layers [25,45,73,100,119].

Fine tuning is also a favoured approach to colour texture classification. It combines the advantages of sophisticated, trusted and high-performance backbone models with reduced demand for computational resources and training data. This practice has been corroborated in a wide range of applications, including sand type classification [120], computer-assisted diagnosis from microscopy [121] and radiographic images [122,123], surface grading [124], identification of plant diseases [125], smoke detection [126] and weed identification [127].

Full training has also been investigated in the context of colour textures; however, the design and training of a general-purpose CNN runs foul of two major issues in this case. First, the semantic gap: whereas other computer vision tasks—such as object classification—rely on unequivocal class labels like ‘coffeepot’, ‘envelope’, ‘fireboat’, ‘crane’, ‘pelican’, etc. (samples of classes featured in the ImageNet dataset [128]), that does not hold true for colour textures. Second, public datasets for colour textures are by orders of magnitude smaller than available for other task (again, object classification is a striking example). As a result, end-to-end fully-trained convolutional networks for colour textures are mostly confined to domain-specific tasks, as for instance classification of histology and microscopy images [129,130], materials recognition [131], defect detection [132] and land-cover classification [133].

## 6. Discussion

In Section 3, Section 4 and Section 5 we have retraced the key concepts that have accompanied the development of colour and texture descriptors during the last half century. There is a major turning point in this history, and that is the appearance and subsequent widespread adoption of deep learning methods starting from 2012. The change in the paradigm that followed was conceptually substantial, shifting the approach to the problem from theory-based to data-driven. The success of deep learning models, usually superior in accuracy to traditional methods (and in some cases even to humans), has led to question whether methods invented before deep learning are still relevant today [134].

Answering this question would lead us outside the scope of this work, we believe, and possibly the times are not mature for an answer. Still, it is important to provide some context and discuss the main advantages and disadvantages of the two approaches. Let’s start with deep learning and its major strengths. First of all, of course, performance. Deep learning can solve some closed-end classification problems with super-human accuracy. Second, in most cases off-the-shelf and/or fine-tuned networks will do the job just fine, with relatively little intervention from the end user. Consequently (third), deep learning often requires less analysis and domain specific knowledge than traditional methods. On the other hand, the major disadvantage is that deep learning is a sort of ‘black-box’ where it is hard to intervene when something goes wrong; mistakes are difficult to detect and correct. This is particularly relevant, for instance, in medical imaging, where accountability issues demand explainable models [135]. For some users the availability of computing power and large enough datasets can also be an issue when it comes to training networks from scratch. Another (minor) inconvenience is that processing free-form images can be complicated, for the receptive field of convolutional networks has fixed shape and size (usually square).

As for hand-designed methods, one major advantage is that they are usually transparent —i.e., produce human-interpretable features. They are also computationally cheap and require little or no training at all. From a performance standpoint, however, they can achieve state-of-the-art accuracy only when the problem is confined within a well defined domain (e.g., steady imaging conditions), otherwise they cannot compete with deep learning.

Interestingly, the theory-driven vs. data-driven debate spreads far beyond the boundaries of computer vision. In a provocative and highly influential paper appeared in 2008 Anderson questioned whether models are actually of any practical use in the era of Big Data [136]. He claimed, in a nutshell, that since data make it possible to establish correlations, we can stop looking for models. Correlations do not of course tell us why certain things happen, but may alert us that they are about to happen and that is just what we need in many applications. There are, however, two major objections to this approach [137]. First, the risk of learning/detecting spurious correlations, which is a well-known problem for instance with convolutional networks [138]. Second, data are never collected ‘randomly’, but always within the framework dictated by methodological and practical constraints. Data-driven approaches certainly represent new opportunities to knowledge discovery, but arguably they will not replace methodological procedures. Rather, theory-driven and data-driven approaches should be seen as complementary steps in the cycle of knowledge discovery [137].

Finally, we would like to emphasize how the theory-based vs. data-driven dichotomy also lies at the heart of one currently ‘hot’ research topic—that is, Explainable Artificial Intelligence (XAI). The main objective of this new research field is to develop models that besides producing accurate results are also understandable by humans [139,140,141]. These methods are actively investigated because explainability has been identified as a major factor in building up trust in algorithmic prescriptions. It is still too early to tell, but perhaps XAI will become the unifying approach of theory- and data-driven descriptors for visual recognition.

## 7. Conclusions

Visual descriptors for colour and texture recognition have long been investigated in computer vision as potential means to reproduce the human capability to discriminate objects, materials and scenes. Starting from the seminal work of Haralick [28] we have reviewed the main ideas and methods that have characterised the development of the field in the last half century. Our overview pivots around two major events: the formalisation of the BoVW model by the turn of the century and the surge of deep networks starting from 2012. The latter, in particular, has brought about a major paradigm change, shifting the approach to the problem from theory-driven to data-driven.

Convolutional networks have the ability to ‘learn’ features from the data, therefore can potentially replace what was essentially a manual activity: feature engineering. The effectiveness of deep networks for colour texture classification has also been confirmed in various studies and the use of CNN (particularly pre-trained and fine-tuned models) is nowadays common practice in a range of applications involving colour textures. Still, this success comes at the cost of lack of transparency: deep networks essentially remain ’black boxes’ to the end users, with their internal logic not explicitly known.

## Figures and Tables

**Table 1:** Some definitions of texture, sorted by order of appearance (newest first).

Definition	Authors, Year	Ref.
A set of texture elements (called texels) which occur in some regular or repeated pattern	Hung, Song and Lan, 2019	[11]
The property of a surface that gives rise to local variations of reflectance	Davies, 2008	[12]
A pattern that can be characterised by its local spatial behaviour and is statistically stationary	Paget, 2008	[13]
The variation of data at scales smaller than the scales of interest	Petrou and García Sevilla, 2006	[14]

**Table 2:** Summary table of CNN architectures.

Name	No. of Weights (≈)	Year	Ref.
AlexNet	62.4 M	2012	[29]
VGG16	138 M	2015	[107]
VGG19	144 M	2015	[107]
GoogLeNet	6.80 M	2015	[108]
ResNet50	25.6 M	2016	[109]
ResNet101	44.7 M	2016	[109]
ResNet152	60.4 M	2016	[110]
DenseNet121	8.06 M	2017	[110]
DenseNet169	14.3 M	2017	[110]
DenseNet201	20.2 M	2017	[110]
MobileNet	4.25 M	2017	[111]
EfficientNetB0–B7	5.33–66.7 M	2019	[112]

## Data Availability

Not applicable.

## Abbreviations

The following abbreviations are used in this manuscript:

BIF	Basic Image Features
BoVW	Bag of Visual Words
BoW	Bag of Words
CIE	Commission Internationale de l’Éclairage
CNN	Convolutional Neural Network(s)
FV	Fisher Vector
GLCM	Grey-Level Co-occurrence Matrices
ICM	Integrative Co-occurrence Matrices
IPBC	Image Patch-Based Classifier
OCLBP	Opponent-Colour Local Binary Patterns
LBP	Local Binary Patterns
RP	Random Projections
RT	Rank Transform
VLAD	Vectors of Locally-Aggregated Descriptors
XAI	Explainable Artificial Intelligence
